# Telehealth in antenatal care: recent insights and advances

**DOI:** 10.1186/s12916-023-03042-y

**Published:** 2023-08-30

**Authors:** Jessica Atkinson, Roxanne Hastie, Susan Walker, Anthea Lindquist, Stephen Tong

**Affiliations:** 1 Department of Obstetrics and Gynaecology, University of Melbourne, Mercy Hospital for Women, 163 Studley Road, Heidelberg, VIC 3084 Australia; 2 Mercy Perinatal, Mercy Hospital for Women, Heidelberg, VIC Australia

**Keywords:** Telehealth, Pregnancy, Antenatal care, Obstetrics, Cost-effectiveness, Maternal outcomes, Neonatal outcomes, Consumer satisfaction, Ambulatory blood pressure monitoring

## Abstract

**Background:**

For decades, antenatal care in high-resource settings has involved 12–14 face-to-face visits across pregnancy. The COVID-19 pandemic forced many care providers to rapidly embrace telehealth to reduce face-to-face visits. Here we review recent advances in telehealth used to provide antenatal care.

**Main body:**

We conducted a narrative review examining the impact of telehealth on obstetric care. Two broad types of telehealth are used in antenatal care. The first is real-time telehealth, where consultations are done virtually instead of face-to-face. The second is remote monitoring, where in-clinic physical examinations are replaced with at-home alternatives. These can include blood pressure monitoring, fetal heart rate monitoring, and emerging technologies such as tele-ultrasound. Large cohort studies conducted during the pandemic era have shown that telehealth appears not to have increased adverse clinical outcomes for mothers or babies. However, further studies may be required to confidently conclude rare outcomes are unchanged, such as maternal mortality, serious morbidity, or stillbirth. Health economic studies suggest telehealth has the potential to reduce the financial cost of care provision. Telehealth in antenatal care seems to be acceptable to both pregnant women and healthcare providers.

**Conclusion:**

Adoption of telehealth technologies may improve the antenatal care experience for women and reduce healthcare expenditure without adversely impacting health outcomes for the mother or baby. More studies are warranted to confirm telehealth does not alter the risk of rare outcomes such as maternal or neonatal mortality.

**Supplementary Information:**

The online version contains supplementary material available at 10.1186/s12916-023-03042-y.

## Background

First developed in the 1800s, antenatal care aims to detect and manage pregnancy complications and monitor the wellbeing of the mother and baby [1, 2]. Prior to this, essentially no additional care was offered to pregnant women and many only sought midwifery or obstetric care at the onset of labour. The introduction of antenatal care was associated with dramatic reductions in maternal and neonatal mortality [3, 4]. Following the widespread implementation of routine antenatal care across the United States in the early 1900s, infant mortality declined by over 90% and maternal deaths fell by 99% [5].

In most high-income settings, the traditional model of antenatal care involves 12–14 face-to-face visits with physical examination [2]. These examinations typically encompass blood pressure monitoring to screen for hypertensive disorders, auscultation of the fetal heart, and measurement of the symphysis fundal height to assess fetal growth (with selective referrals for ultrasound).

In 2020, the onset of the COVID-19 pandemic forced many medical specialties to rethink their approach to outpatient care in a bid to reduce face-to-face contact. This instigated a rapid shift towards telehealth [6–8]. In antenatal care, telehealth has various hypothetical benefits, including reduced economic burden and increased system efficiency [9]. Importantly, it may be more convenient for pregnant women [9]. However, the use of telehealth replaces direct physical examination, and this raises safety concerns — it may result in a lesser quality of clinical care which puts women at increased risk of adverse pregnancy outcomes [10]. It is therefore possible that the rapid implementation of telehealth could lead to complications being missed (or diagnoses delayed), and an increased rate of adverse outcomes.

There are no comprehensive, up-to-date reviews of the impact of telehealth on antenatal care in high-income settings. This review aims to bridge this gap and provide a narrative overview of a topical subject. We conducted a search of PubMed and MEDLINE in March 2023 for the key words ‘antenatal’, ‘obstetrics’, ‘prenatal’, ‘maternity care’, ‘telehealth’, and ‘telemedicine’. Databases were searched from inception until 10 March 2023. Our search identified 7048 papers. After excluding duplicate records, 5125 papers were manually screened for inclusion. Complete search strategies are included in Additional file 1: Table S1 and Additional file 2: Table S2. We aimed to review the contemporaneous shift to telehealth with the emergence of the COVID-19 pandemic. Therefore, we focused our literature search primarily (but not exclusively) on papers published between January 2020 and March 2023.

Papers were considered for inclusion if they provided data or commentary on one of the following aspects of telehealth in antenatal care: models of telehealth, clinical safety, cost-analyses, or consumer satisfaction. Papers were grouped according to their theme(s). Results were synthesised based on overarching trends, which became evident through our review of the literature.

We first describe the different telehealth technologies available for antenatal care. We then review the literature on safety — whether the use of telehealth impacts the risk of adverse pregnancy outcomes. We finally examine health economic analyses and studies assessing the acceptability of telehealth to pregnant women and healthcare providers.

## Models of telehealth in antenatal care

Telehealth in antenatal care can be classified into two categories: real-time and remote monitoring. ‘Real-time’ telehealth is designed to replace some (but not all) face-to-face consultations and involves phone calls or video conversations between the pregnant woman and their clinician [11]. Remote monitoring involves the use of technology to replace aspects of the physical examinations that occur during clinic visits, including some fetal monitoring and investigations. The most basic types are blood pressure monitoring and recording the fetal heart rate (via home Doppler) [12]. There are also more advanced remote monitoring techniques used to replace investigations, such as ‘at home’ cardiotocograph monitoring and even an emerging technology called ‘tele-ultrasound’ to replace in-person ultrasound assessment [12, 13].

Currently, the American College of Obstetricians and Gynecologists (ACOG) is the only major obstetric authority to have published guidance on the models of telehealth. ACOG defines telehealth models as either ‘synchronous’ (equates to real-time), ‘asynchronous’ (sending medical imaging to specialists for later interpretation), or remote monitoring [14].

### Real-time telehealth

The COVID-19 pandemic accelerated the uptake of real-time telehealth in antenatal care [15–21]. The general approach has been to continue the existing number of antenatal visits (12–14 appointments) but replace a variable number of in-person visits with virtual ones [15–21]. In most models, face-to-face visits are scheduled at key gestations across pregnancy. This typically includes the first visit, at 28 weeks, and at 36 weeks’ gestation (these are milestone visits, where there is often more involved planning for pregnancy and birth than other visits). Below we describe two models to provide examples, but many variations have been reported (see Table 1).

**Table 1:** Models of telehealth in obstetrics published since 2020

**Author**	**Centre**	**Population**	**Model**	**Remote monitoring**	**Proposed number of visits—in-person**	**Proposed number of visits—telehealth**
Aziz et al. [15]	Columbia University Irving Medical Centre (USA)	High-risk pregnancies	Based on existing antenatal care model; supplementing face-to-face visits with virtual visits conducted with online videoconferencing software	Yes**—**ambulatory blood pressure, remote glucose monitoring.	6 in-person visits (11–13wk; 18–22wk; 27–28wk; 36wk; 39wk; 40wk)	7–8 telehealth visits (intake; 11–14wk; 23–26wk; 29–31wk; 32–35wk; 37wk; 38wk)
Dosaj et al. [16]	University of Illinois at Chicago (USA)	Low-risk pregnancies	Based on existing antenatal care model; supplementing face-to-face visits with virtual visits conducted with online videoconferencing software	Yes—ambulatory blood pressure as indicated, fetal Doppler as indicated	7 face-to-face visits (12wk; 20wk; 28wk; 32wk; 36wk; 38wk; 40wk)	6+ telehealth visits (intake; 12–28wk as necessary; 30wk; 34wk; 37wk; 39wk)
Duryea et al. [22]	Parkland Hospital, Dallas, TX (USA)	Low-risk and high-risk pregnancies	Based on existing antenatal care model; supplementing face-to-face visits with virtual visits conducted with online videoconferencing software	No	10 face-to-face visits (10wk; 18–20wk; 24wk; 28wk; 32wk; 36wk; 38wk; 39wk; 40wk; 41wk)	3 telehealth visits (14wk; 34wk; 37wk)
Fryer et al. [18]	Hillsborough County, FL (USA)	Low-risk pregnancies	The ‘OB Nest Model’	Yes—ambulatory blood pressure, remote fetal Doppler	6 face-to-face visits (10–14wk; 20-22wk; 27–28wk; 35–36wk; 39wk; 40–41wk)	5 telehealth visits (6–10wk; 15–19wk; 23–26wk; 29–34wk; 37–38wk)
Limaeye et al. [23]	NYU Langone Health (USA)	Low-risk and high-risk pregnancies	Based on existing antenatal care model; supplementing face-to-face visits with virtual visits conducted with online videoconferencing software	No	6 face-to-face visits (11–14wk; 20–22wk; 27–28wk; 36wk; 38wk; 40wk)	7 telehealth visits (6–10wk; 15–19wk; 23–26wk; 29–31wk; 32–35wk; 37wk; 39wk)
Nakagawa et al. [21]	Hokkaido University Hospital (Japan)	Low-risk and high-risk pregnancies	Based on existing antenatal care model; supplementing face-to-face visits with virtual visits conducted with online videoconferencing software	Yes—ambulatory blood pressure, remote cardiotocography	5 face-to-face visits (12wk; 20wk; 24wk; 30wk; 36wk)	Variable number of telehealth visits (all other appointments as needed)
Palmer et al. [20]	Monash Health (Australia)	Low-risk pregnancies	Based on existing antenatal care model; supplementing face-to-face visits with virtual visits conducted with online videoconferencing software	Yes—ambulatory blood pressure, self-measured symphysial fundal height	3 face-to-face visits (28wk; 36wk; ≥ 40wk)	6 telehealth visits (intake; 16wk; 22wk; 31wk; 34wk; 38wk)
Palmer et al. [20]	Monash Health (Australia)	High-risk pregnancies	Based on existing antenatal care model; supplementing face-to-face visits with virtual visits conducted with online videoconferencing software	Yes—ambulatory blood pressure, self-measured symphysial fundal height	5 face-to-face visits (16–18wk; 28wk; 36wk; 38wk; ≥ 40wk)	5 telehealth visits (intake with midwife; intake with obstetrician; 22wk; 31wk; 34wk)
Peahl et al. [17]	University of Michigan (USA)	Low-risk pregnancies	The ‘4-1-4 Model’	No	5 face-to-face visits (8wk; 19wk; 28wk; 36wk; 39wk)	4 telehealth visits (16wk; 24wk; 38wk; 38wk)
Tavener et al. [19]	Imperial College Healthcare NHS Trust (UK)	Low-risk pregnancies	Based on existing reduced antenatal care model; supplementing face-to-face visits with virtual visits conducted with online videoconferencing software	No	8 face-to-face visits (14wk; 20wk; 28wk; 32wk; 36wk; 38wk; 40wk; 41wk)	2 telehealth visits (intake; 16wk)

**Fig. 1:**
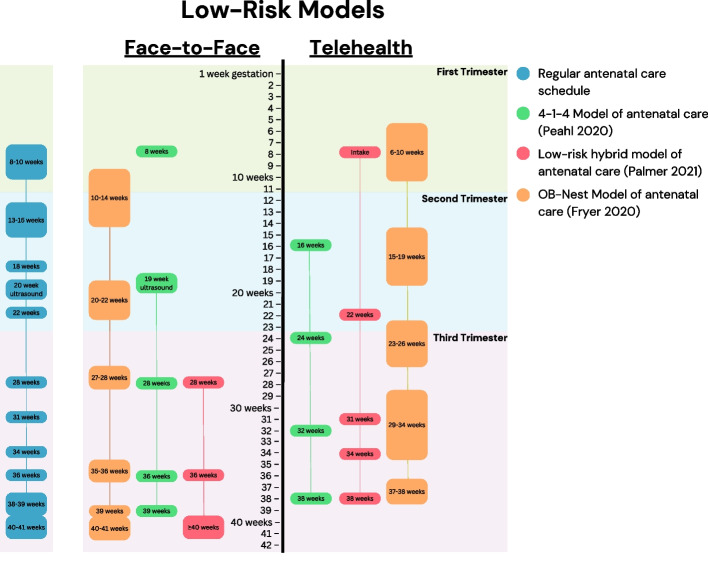
Schedule of antenatal visits for low-risk antenatal telehealth models. Schedule of face-to-face and telehealth visits for three antenatal telehealth models for low-risk pregnancies (Peahl et al. [17], in green; Palmer et al. [20], in red; Fryer et al. [18], in orange), compared with the standard antenatal care schedule (in blue)

Peahl et al. [17] described the implementation of the 4-1-4 model at the University of Michigan, tailored for low-risk pregnancies [17]. This model involves 4 in-person visits (the first visit at 8 weeks, then 28, 36, and 39 weeks’ gestation), 1 ultrasound (19 weeks’ gestation), and 4 virtual visits (at 16, 24, 32, and 38 weeks’ gestation) (Fig. 1). The authors note that pregnant women and their practitioners appreciated the benefits of a hybrid model [17].

**Fig. 2:**
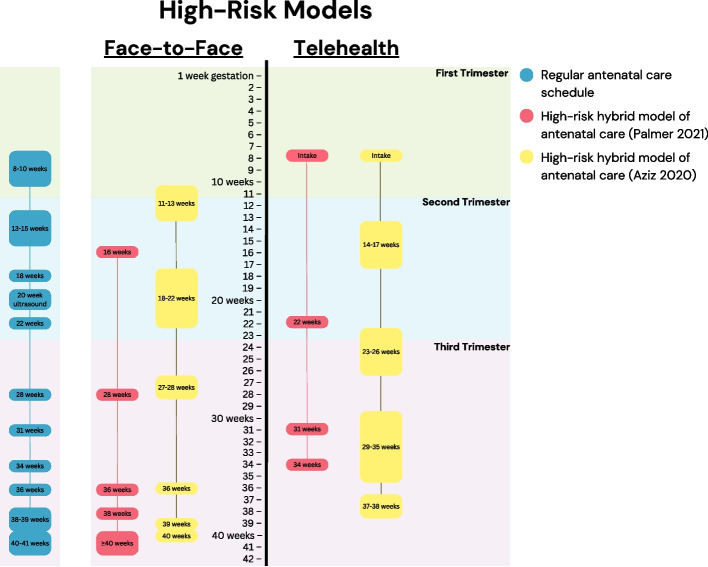
Schedule of antenatal visits for high-risk pregnancy telehealth models. Schedule of face-to-face and telehealth visits for two antenatal telehealth models for high-risk pregnancies (Palmer et al. [20], in red; Aziz et al. [15], in yellow), compared with the standard antenatal care schedule (in blue)

Palmer et al. [20] implemented a similar model. The team devised protocols with a different number of telehealth visits for low-risk (midwifery-led, shared care, or collaborative care) and high-risk pregnancies (obstetric specialist-led). Their low-risk model involves 6 telehealth (at intake, then 16, 22, 31, 34, and 38 weeks’ gestation) and only 3 in-person visits (at 28, 36, and ≥ 40 weeks’ gestation) (Fig. 1). Their high-risk model involves 5 telehealth (midwifery intake, obstetrician intake, and 22, 31, and 34 weeks’ gestation) and 5 in-person visits (at 16, 29, 36, 38, and ≥ 40 weeks’ gestation) (Fig. 2) [20]. While these models were initially developed to accommodate COVID-19 social distancing requirements, the authors note that they will be used at their centre beyond the pandemic due to their success [20].

### Remote monitoring

**Fig. 3:**
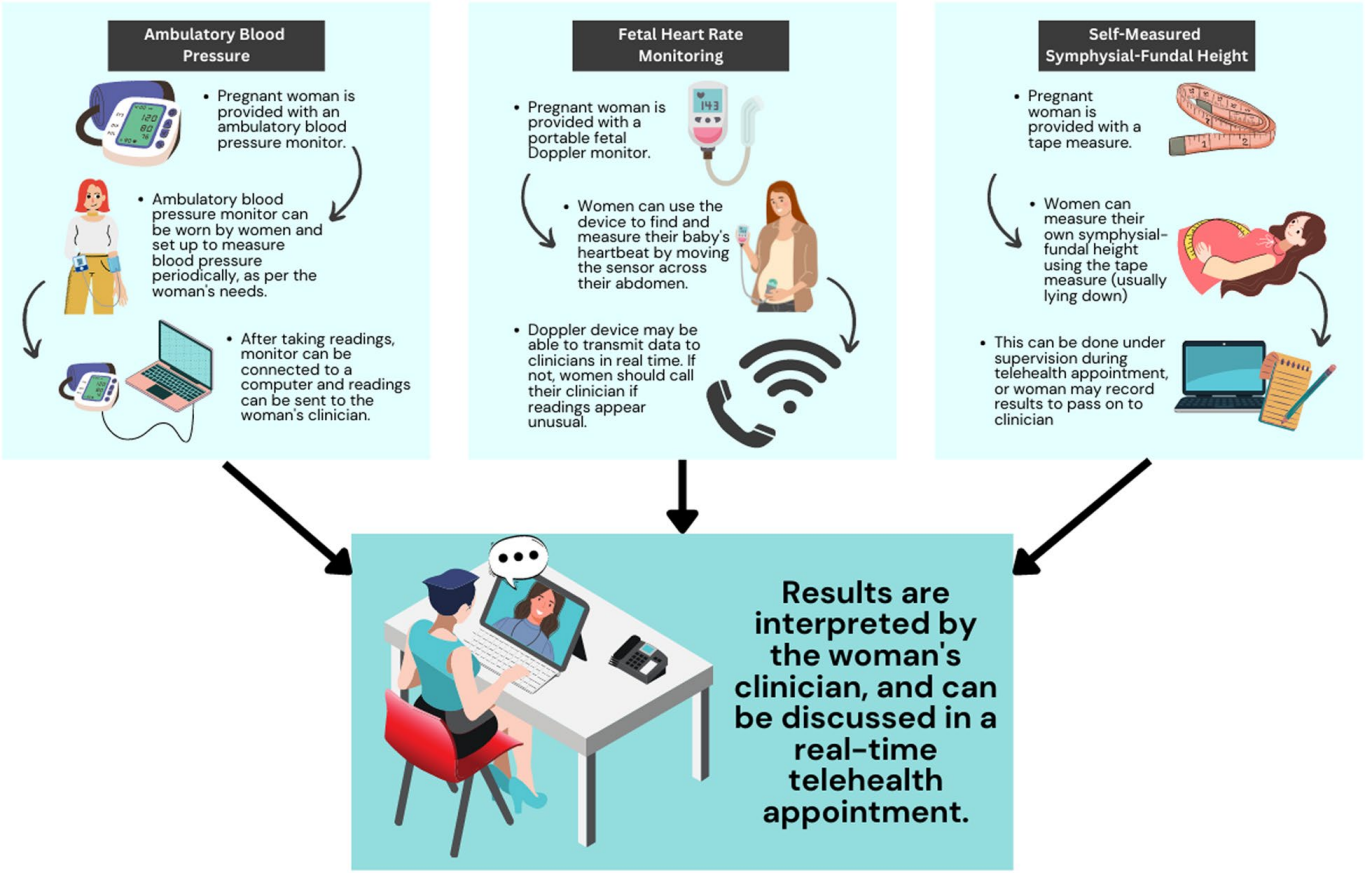
Summary of the main remote monitoring technologies currently used in antenatal care

We also have the option to integrate remote monitoring technologies to replace direct physical examination, so the entire consultation can be virtual. Remote monitoring is made possible through the use of wearable or portable devices. These collect health and biometric data from pregnant women which are transmitted to their healthcare providers (Fig. 3). Remote monitoring of various parameters such as blood pressure, fetal heart rate, and fetal growth (via tape measure) has been shown to be as accurate as in-clinic assessment by the health care provider [15, 18, 20, 21].

#### Remote blood pressure monitoring

At-home blood pressure monitoring (or ambulatory blood pressure monitoring) is used routinely by other disciplines and may be better than in-clinic monitoring at detecting masked hypertension and reducing white-coat hypertension [24]. This is because it side-steps ‘white-coat hypertension’, where the blood pressure is artificially elevated from baseline due to the stress of visiting the clinic.

A systematic review published in 2018 suggested there was little difference between in-clinic and at-home blood pressure readings during pregnancy [25]. Recent large trials have reported similar findings. In a secondary analysis of the OPTIMUM-BP randomised trial (which compared self-monitoring of blood pressure with standard care in the United Kingdom), Bowen et al. [26] showed that 91 women with gestational hypertension or preeclampsia could successfully record their own blood pressure with little variation from in-clinic readings [27]. In the BUMP 2 randomised trial, which included 850 pregnant women, Chappell et al. [28] concluded that blood pressure readings in the home monitoring and usual care (clinic readings) groups were similar.

#### Fetal heart rate monitoring

Monitoring the fetal heart rate by Doppler, or fetal heart rate patterns by cardiotocography, may also be performed remotely. Porter et al. [29] demonstrated the utility of HeraBEAT, a handheld fetal Doppler device which can be used by women to find and measure their baby’s heartbeat and transmit data to clinicians in real time. The device uses a smartphone interface to guide women on its use. Porter et al. demonstrated that data from these devices were equivalent to those recorded in clinics [29]. They suggest that this would allow these devices to be used in telehealth consultations, in place of clinician measurement. The HeraBEAT device is already available to be used in the clinic [30, 31].

However, concerns have been raised about listening to the fetal heart rate at home. It has been suggested that in the situation of reduced fetal movements, women may be falsely reassured by simply detecting a fetal heartbeat [32]. This may falsely reassure women and stop them from urgently seeking further care [32]. In such situations, detecting a fetal heartbeat is not sufficient: women should have a full cardiotocograph assessment to more confidently rule out acute fetal hypoxia [33].

The HeraBEAT device may also be used to record continuous traces over several minutes, with comparable results to cardiotocographs done at a hospital visit [29]. Other remote cardiotocograph devices are currently on the market [34–36]. One such device, the iCTG from Melody International, has been shown accurately to monitor fetal heart rate patterns in pregnancies complicated by fetal growth restriction [36]. In the recently published HoTeL trial, Bekker et al. [37] randomised 201 high-risk women to hospital care or remote monitoring (at-home cardiotocography using the Sense4Baby system [ICT Healthcare Technology Solutions, Netherlands] and blood pressure monitoring). Bekker et al. demonstrated that remote monitoring was non-inferior to hospital admission for high-risk women (including pregnancies complicated by preeclampsia, fetal growth restriction, preterm rupture of membranes, gestational diabetes, fetal anomalies, and imminent preterm birth) [37].

Given the different indications for fetal Doppler and cardiotocograph, women should receive individualised counselling on using these devices and use them primarily during telehealth appointments under clinician supervision [32].

#### Self-measured symphysial fundal height

Palmer et al. [20] employed self-measured symphysial-fundal height supported by educational material within their telehealth model. Bergman et al. [38] had previously demonstrated the feasibility of this technique. Bergman et al. found there was greater individual variance in measurements from pregnant women than midwives, but this could be overcome by asking women to take multiple measurements at each visit [38]. During the telehealth period, Palmer et al. saw a rate of undetected fetal growth restriction of 24% for low-risk pregnancies and 5% for high-risk pregnancies. This was not significantly different from the conventional care period (24% low-risk and 11% high-risk), which indicates that self-measured symphysial-fundal height may be as accurate as in-clinic measurements in detecting poor fetal growth [20].

#### Tele-ultrasound

**Fig. 4:**
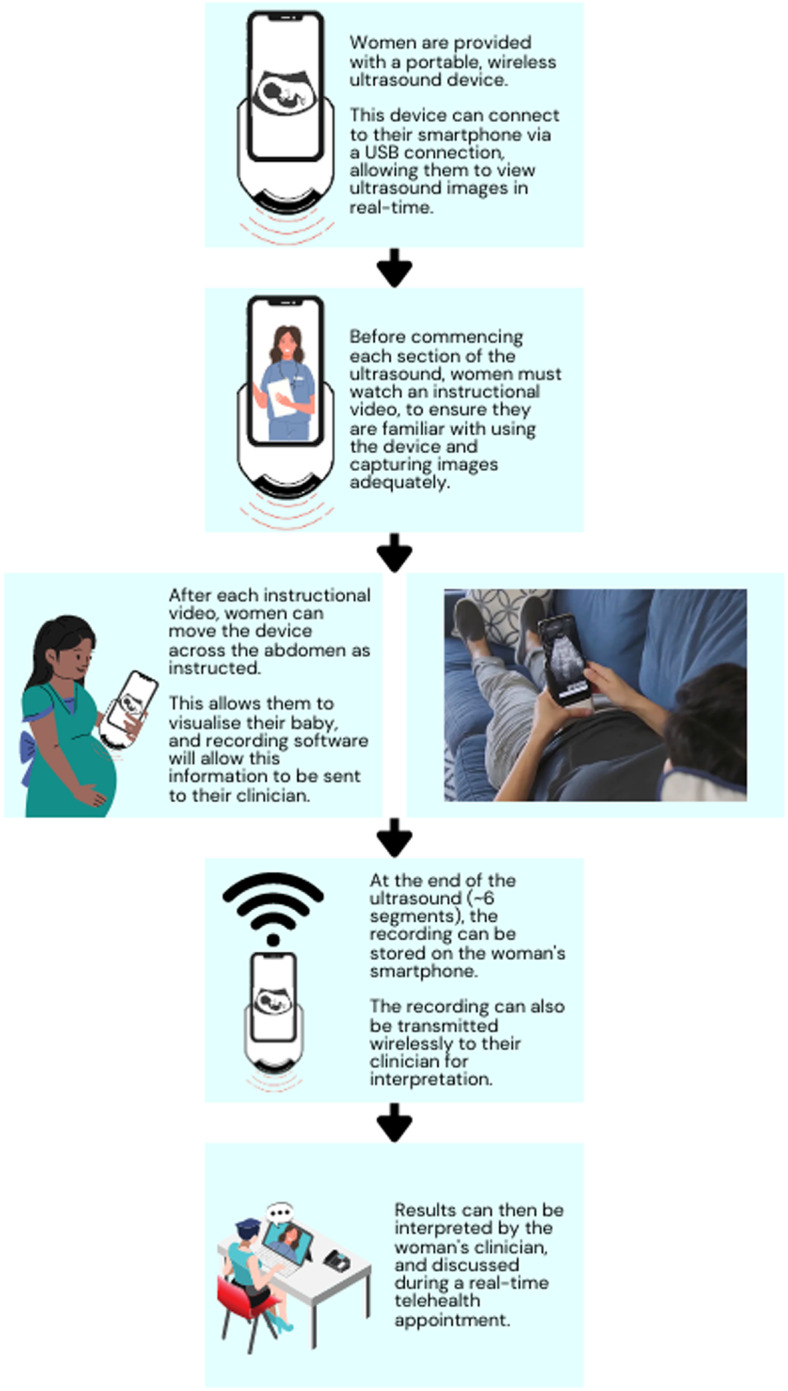
Self-recorded tele-ultrasound (adapted from Hadar et al. [40])

Emerging technology is now allowing ultrasound examinations to be conducted remotely. Known as tele-ultrasound, this technology was previously used in rural areas so inexperienced sonographers could be supervised remotely [39]. In recent years, trials have explored self-operated tele-ultrasound, where women perform ultrasounds on themselves at home and transmit the data to their clinical team (see Fig. 4).

Hadar et al. [40] recently performed an observational study of tele-ultrasound, supplying women with self-operated tele-ultrasound devices to assess their fetus’ biophysical profile. Components of the biophysical profile include parameters such as fetal tone, breathing, movement, and amniotic fluid volume, which are captured by ultrasound. An abnormal biophysical profile has a strong association with many adverse perinatal outcomes (suggesting a low score reflects poor fetal health) [41].

Hadar et al. used the *INSTINCT* ultrasound device, developed by PulseNmore (Omer, Israel) [40]. The device attaches to the woman’s mobile phone so they can see the ultrasound images on their screen and transmit videos to their clinicians [40]. Women perform their first ultrasound under the guidance of an experienced technician. They perform the scan in six segments to measure the biophysical profile — each accompanied by an instructional video telling women where to place the device and how to move it across the abdomen [40]. Women can perform these scans at home to monitor fetal wellbeing as often as clinically directed.

Among 100 women undertaking 1360 scans, the fetal heart activity was detected successfully 95.3% of the time [40]. Success in detecting each component of the biophysical profile varied: normal amniotic fluid volume (92.2%), body movements (88.3%), fetal tone (69.4%), and fetal breathing movements (23.8%) [40, 41]. Our search identified no studies utilising patient-operated tele-ultrasound to estimate fetal weight, which may be an avenue for future research and development.

## Clinical safety

It is important to examine safety outcomes when replacing face-to-face visits and physical examinations with telehealth. There is concern that this change in practice may compromise care, particularly if diagnoses are delayed or missed. For example, missing diagnoses of preeclampsia or fetal growth restriction could lead to rare but serious adverse maternal and perinatal outcomes, such as eclampsia, maternal morbidity or mortality, and stillbirth [42–44]. Appropriately powered studies are necessary to ensure that the implementation of telehealth does not compromise maternal or neonatal safety.

### Obstetric outcomes

#### Preeclampsia and gestational hypertension

In a meta-analysis comparing telehealth (both remote monitoring and real-time) with conventional care among women with high-risk pregnancies, no increased risk of hypertension, preeclampsia, or eclampsia was found (odds ratio [OR] 0.90 [95% confidence interval (CI) 0.62, 1.29], *p* = 0.59; *N* = 1 285) [45].

In their large, retrospective analysis of 22,323 pregnancies, Palmer et al. [20] compared conventional care (pre-COVID-19) with real-time telehealth and remote monitoring (during COVID-19). Reassuringly, they observed no difference in rates of preeclampsia for low-risk (3% vs 3%, *p* = 0.70) or high-risk (7% vs 9%, *p* = 0.15) women between conventional care and telehealth periods [20]. The authors also completed a follow-up analysis after 12 months of telehealth in their centre and again reported no difference in rates of preeclampsia [46].

Similarly, Duryea et al. [22] conducted a cohort study of 12,067 women and compared pre-pandemic conventional care with pandemic-era telehealth (audio-only visits). They saw a slight decrease in rates of gestational hypertension (adjusted risk ratio [aRR] 0.93 [95% CI 0.86, 0.99], *p* = 0.10) but no change in rates of severe preeclampsia (aRR 0.99 [95% CI 0.89, 1.09], *p* = 0.85) [22].

Taken together with the studies on the accuracy of home blood pressure monitoring, it seems the literature reassuringly suggests monitoring blood pressure at home does not compromise the timely diagnosis of preeclampsia.

#### Fetal growth restriction

In their meta-analysis of 230,000 high-risk pregnancies, Güneş Öztürk et al. [45] noted no increase in the rates of fetal growth restriction among women who received telehealth (real-time or remote monitoring) compared with conventional care. However, only two included studies reported on this outcome and numbers were therefore small (OR 1.46 [95% CI 0.59, 2.91], *p* = 0.51;* N* = 305). Palmer et al. [20] also observed no difference in rates of fetal growth restriction (birthweight < 10th centile) in low-risk (10% vs 10%, *p* = 0.71) or high-risk (14% vs 13%, *p* = 0.55) pregnancies after implementing telehealth, which included self-measured symphysial fundal height.

Importantly, Palmer et al. saw no difference in the rates of undetected fetal growth restriction in their initial analysis (defined as the proportion of babies born with a birthweight < 3rd centile after 40 weeks’ gestation, compared with all babies born <3rd centile after 32 weeks' gestation) [20]. They also observed no difference in the rates of undetected fetal growth restriction during their 12-month follow-up analysis for low-risk (20.6% telehealth vs 28.6% conventional care, *p* = 0.12) or high-risk (8.5% vs 10.2%, *p* = 0.72) models [46]. In a retrospective study of 2641 women, Soffer et al. [47] similarly found no change in the rates of undetected fetal growth restriction (defined as an infant <10th centile without an antenatal diagnosis of fetal growth restriction) (61.7% conventional care vs 64.4% telehealth by phone or video, *p* = 0.76). Soffer et al. also noted that the median age of diagnosis did not differ between conventional care (36 weeks) and telehealth (37 weeks, *p* = 0.44) [47].

Hence, large cohort studies have reassuringly concluded replacing intensive clinic visits to measure symphysial-fundal height with telehealth has not led to increased rates of undiagnosed fetal growth restriction.

#### Preterm birth

Güneş Öztürk et al. reported no increased risk of preterm birth across five studies (OR 0.65 [95% CI 0.38, 1.13], *p* = 0.13; *n* = 928) [45]. In a systematic review (without meta-analysis), Ghimire et al. [48] reported no increased risk in the rates of preterm birth from four studies examining real-time telehealth. In the HoTeL randomised trial, Bekker et al. [37] reported no increased risk of preterm birth among high-risk pregnancies utilising remote monitoring, compared with in-hospital monitoring (risk difference [RD] −0.090 [95% CI −0.225, 0.045], *N* = 201).

Although evidence is limited, some studies have indicated that telehealth may even be associated with a decreased risk of preterm birth. In their interrupted time-series model, Palmer et al. saw a significant reduction in preterm birth among high-risk pregnancies (0.68% reduction each week [95% CI −1.37%, −0.002%], *p* = 0.049) [20]. This association was not present in their main analysis, or among low-risk pregnancies [20]. The association was also not present in their 12-month follow-up [46]. Duryea et al. observed a similar trend when stratifying by number of telehealth visits (10.2% conventional care; 9.1% with 1 telehealth visit; 7.1% with 2 telehealth visits; 8.1% with 3 telehealth visits; *p* < 0.001) [22].

It is important to note that both Palmer et al. and Duryea et al. conducted their studies during the COVID-19 pandemic and compared outcomes with pre-pandemic data. It has been established that COVID-19 lockdown measures were associated with reduced preterm birth rates, which may account for the reductions seen [49, 50]. However, in their pre-pandemic meta-analysis of women with gestational diabetes, Xie et al. [51] also found that telehealth was associated with a decreased incidence of preterm birth (risk ratio [RR] 0.27 [95% CI 0.20, 0.35], *p* < 0.01) [51].

The reasons for this association, if true, remain unclear. Delayed diagnosis and reduced intervention for suspected maternal or fetal compromise is a plausible contributor to a reduction in iatrogenic preterm birth [50]. This highlights the importance of including all potential downstream outcomes (e.g. stillbirth), when evaluating the overall safety and efficacy of telehealth.

#### Diabetes

Among diabetic women, telehealth is associated with similar, or even improved, outcomes. In their meta-analysis of 5108 women with gestational diabetes, Xie et al. showed that telehealth (real-time or remote monitoring) was associated with improved glycated haemoglobin (HbA1c) (mean difference [MD] −0.70% [95% CI −1.05, −0.34], *p* < 0.01), reduced 2-h postprandial blood glucose (MD −1.03mmol/L [95% CI −1.83, −0.23], *p* = 0.01), and reduced fasting blood glucose (MD −0.52mmol/L [95% CI −0.81, −0.24], *p* < 0.01) [51]. Diabetic women receiving telehealth also had lower rates of caesarean section, premature rupture of membranes, preeclampsia, and polyhydramnios [51]. Systematic reviews from Germany, the UK, and Singapore have also shown improved HbA1c control among women who used telehealth, while a systematic review from Denmark showed no difference in maternal outcomes [52–55].

In their 12-month follow-up analysis, Palmer and colleagues saw that significantly more women were diagnosed with gestational diabetes during the telehealth period (25.1% low risk; 34.0% high risk) compared with conventional care (22.2% low risk [*p* < 0.001]; 28.7% high risk [*p* < 0.001]) [46]. However, there were no differences in the rates of large for gestational age babies, or the number of women treated with insulin [46]. COVID-19 pandemic restrictions in Melbourne (the setting of the study by Palmer et al. [46]) have also been shown to be associated with increased prevalence of gestational diabetes, and this possibly accounts for their findings [56].

#### Caesarean section

Güneş Öztürk et al. found that telehealth was associated with a non-significant reduction in emergency caesarean section rates (OR 0.54 [95% CI 0.29, 1.00], *p* = 0.05; *n* = 648) [45]. Ghimire et al. reported no increase in caesarean section from four studies, and Duryea et al. found no significant difference in caesarean section rates when adjusting for race and body mass index in their study (aRR 1.03 [95% CI 0.98, 1.09], *p* = 0.03) [22, 48]. However, Duryea et al. did report an increase in the rate of primary caesarean section (aRR 1.10 [95% CI 1.01, 1.21], *p* = 0.01) [22]. In the HoTeL randomised trial, Bekker et al. reported no increased risk of caesarean section during labour (RD −0.010 [95% CI −0.108, 0.088], *n* = 201), when comparing telemonitoring (blood pressure and cardiotocograph) with hospital admission [37].

#### Birth interventions

Güneş Öztürk et al. reported that high-risk women who received telehealth were more likely to undergo labour induction — 48.5% in the telehealth group compared with 35.2% in the control group (OR 1.94 [95% CI 1.26, 2.99], *p* = 0.003; *N* = 356) [45]. While the OR seems high, only two studies reported on this outcome, so numbers were small. They also reported that women had no increased risk of instrumental birth (OR 1.44 [95% CI 0.95, 2.18], *p* = 0.09; *N* = 1020) or episiotomy (OR 1.04 [95% CI 0.49–2.20], *p* = 0.93; *N* = 244) [45]. Duryea et al. reported no difference in the rates of spontaneous vaginal birth in their cohort (aRR 0.99 [95% CI 0.96, 1.01], *p* = 0.11) [22].

#### Birth complications

In their meta-analysis, Güneş Öztürk et al. reported no increased risk of shoulder dystocia (OR 4.13 [95% CI 0.46, 37.34], *p* = 0.21; *N* = 607), major perineal trauma (OR 3.00 [95% CI 0.31, 29.48], *p* = 0.35; *N* = 278), premature rupture of membranes (OR 0.88 [95% CI 0.45, 1.72], *p* = 0.71; *N* = 268), or postpartum haemorrhage (OR 0.53 [95% CI 0.05, 6.15], *p* = 0.61; *N* = 69) for women receiving telehealth [45]. Duryea et al. saw no difference in the incidence of postpartum haemorrhage (aRR 1.04 [95% CI 0.93, 1.16], *p* = 0.26) or hysterectomy (aRR 0.53 [95% CI 0.27, 1.04], *p* = 0.07) [22]. In fact, they reported a significant decrease in the rate of shoulder dystocia (aRR 0.48 [95% CI 0.26–0.91], *p* = 0.02) [22].

#### Maternal mortality

Perhaps because of the rarity of maternal mortality in high-income settings, few studies have reported on this outcome [57]. A single study of 228,349 high-risk pregnancies from Hangzhou, China, reported that telehealth (online education) resulted in a lower maternal mortality rate, compared with standard care (4.19 per 100,000 vs 5.19 per 100,000; *p* < 0.05) [58]. This telehealth model included online education and advice and was conducted before the COVID-19 pandemic [58]. We did not identify any other studies from high-income settings examining this outcome, highlighting a need for further research.

### Fetal and neonatal outcomes

#### Fetal parameters

Reported fetal and neonatal outcomes are generally unchanged with telehealth. In their meta-analysis, Güneş Öztürk et al. demonstrated no difference in fetal parameters such as small for gestational age, large for gestational age, or fetal macrosomia [45]. Palmer et al. also reported no difference in rates of macrosomia in low-risk diabetic (11% vs 9%, *p* = 0.10) or high-risk diabetic (16% vs 17%, *p* = 0.79) pregnancies [20]. Bekker et al. also reported no increased risk of small for gestational age or congenital anomalies [37].

#### Neonatal outcomes

Güneş Öztürk et al. showed no difference between telehealth and conventional care for neonatal complications. These included low 5-min Apgar score < 7 (OR 0.54 [95% CI 0.14, 2.14], *p* = 0.38; *N* = 236], respiratory distress syndrome (OR 0.65 [95% CI 0.38, 1.13], *p* = 0.99; ***N***= 928), hypoglycaemia (OR 1.18 [95% CI 0.74, 1.87], *p* = 0.48; *n* = 701), and hyperbilirubinemia (OR 0.86 [95% CI 0.48, 1.54], *p* = 0.61; *N* = 583) [45]. Other studies have supported these findings, in addition to no increased risk of neonatal asphyxia or low umbilical cord pH [20, 22, 37, 45, 59–62].

Palmer et al. saw no increased risk of Neonatal Intensive Care Unit (NICU) admission among low-risk pregnancies (2% vs 2%, *p* = 0.60); however, there was an increased risk among high-risk pregnancies receiving telehealth (15% vs 18%, *p* = 0.01) [20]. Interestingly, this result was reversed in their 12-month follow-up, with NICU admission declining after telehealth integration [46]. Duryea et al. showed no difference in the rates of NICU admission among full-term infants (aRR 1.03 [95% CI 0.78, 1.36], *p* = 0.78), and Güneş Öztürk et al. demonstrated no difference in NICU or special care nursery admissions [22, 45].

#### Stillbirth and neonatal mortality

There are conflicting reports on the impact of telehealth on perinatal deaths. These events are rare, so small numbers preclude confident risk assessment. Duryea et al. reported no increased rate of stillbirth with virtual care (aRR 0.80 [95% CI 0.50, 1.29], *p* = 0.32) [22]. However, the overall incidence was low (29 [0.5%] in the telehealth group vs. 40 [0.6%] in the conventional care group) [22]. Palmer et al. also saw no increase in rates of stillbirth, but were again limited by low incidence (11 [1%] in the telehealth group vs. 105 [1%] in the conventional care group) [20]. They also observed no difference in overall stillbirth rates in their follow-up analysis (0.78% vs 0.81%, *p* = 0.81) [46].

In a state-wide analysis from Victoria, Australia, during the first year of the COVID-19 pandemic, Hui et al. [50] found an increased risk of preterm stillbirth (adjusted odds ratio [aOR] 1.49 [95% CI 1.08, 2.05], *p* = 0.015; *N* = 74,834 births). Of note, there were also reduced iatrogenic preterm births during COVID lockdown in Melbourne (Victoria), Australia [50]. It is plausible then that these observations are explained by the fact that missed diagnoses reduced timely iatrogenic birth to prevent cases of stillbirth. Although they did not examine the effect of telehealth specifically, there was a rapid increase in antenatal telehealth in Australia during the study period [50]. This potential association warrants further investigation.

In a retrospective analysis of 400 high-risk pregnancies, Zizzo et al. [63] reported no neonatal deaths attributable to telehealth (all were secondary to other causes). Güneş Öztürk et al. saw no increased risk of neonatal mortality (OR 0.69 [95% CI 0.17, 2.77], *p* = 0.60; *N* = 228,469); however, this result was heavily influenced by a single population study [45, 58]. Given the rarity of stillbirth and neonatal mortality in high-resource settings, further investigation is warranted.

In summary, the studies thus far have not found telehealth is associated with increased risks of adverse obstetric or neonatal events. More studies are required to investigate rare outcomes.

## Cost-effectiveness

### Global cost-benefit

There are few studies on the cost-effectiveness of antenatal telehealth. However, existing research has been generally positive. Van den Heuvel et al. [64] conducted a cost-analysis of their study involving a digital health platform and remote monitoring to reduce face-to-face antenatal visits for women at increased risk of preeclampsia. They found their telehealth model was associated with an average saving of 19.7% (USD $844.30 per woman) when compared with traditional antenatal care [64].

In their randomised trial, Bekker et al. [37] found significant cost-benefit. High-risk women were randomised to receive remote monitoring (blood pressure and cardiotocography) or hospital admission. The mean total cost per participant for the remote monitoring group was $20,393 USD, compared with $28,459 USD for the hospital group [37]. This decline in costs was largely driven by the significant reduction of admitted days for the remote monitoring group.

Among pregnant women with pre-existing diabetes, Sung et al. [65] demonstrated that telehealth, compared with in-person antenatal care, saved an average of $2,798 per woman. While Theiler et al. [66] found that the OB-Nest reduced-visit model (8 in-person visits and 6 virtual nursing visits) was associated with higher nursing costs, the overall cost to providers was reduced. Cost analyses of telehealth in other medical specialties have all pointed towards cost-benefits [67, 68].

## Consumer satisfaction

### Patient satisfaction

Satisfaction with antenatal telehealth among pregnant women has been consistently high across various studies. In a recent systematic review, Konnyu et al. [69] examined 251 pregnant women’s experiences with telehealth. The authors identified several themes in their analysis, including concerns that telehealth may lead to less timely information, concerns about taking on more personal responsibility with reduced visit schedules, and concerns about safety [69]. However, they also reported that women believed telehealth could be tailored to suit their needs better than traditional care [69].

Ghimire et al. [48] conducted a much larger systematic review of women’s experiences. Their review included 23 studies from 2011 to 2021 with over 15,000 pregnant women [48]. They identified that women preferred video conferencing over telephone; that women preferred communicating in their own language; that technology needed to be straightforward, flexible, and user-friendly; and that women felt telehealth should be cheaper than traditional care [48]. Women showed a strong preference towards a mixed model of in-person and virtual visits, and multiparous women preferred virtual care [48].

Ghimire et al. identified several barriers to care, including a lack of consistent and high-speed Internet; low technology literacy; language challenges; privacy concerns; and lack of empathy [48]. Other studies have also noted difficulties with technology and lack of connection with clinicians as limitations of telehealth [70–83]. Enablers identified by Ghimire et al. included increased access to care; reduced absence from work/reduced travel time; increased self-management skills; cost-benefits; minimised exposure to COVID-19 pandemic; and increased confidence and connection [48].

In their observational study of tele-ultrasound, Hadar et al. [40] examined 100 women’s experience with the service. Women were asked to complete a questionnaire after the study, and the average rating for user experience was 4.4/5 (standard deviation [SD] 0.6), while the average rating for user satisfaction was 3.9/5 (SD 1.2), indicating that women were generally satisfied with the service and found it easy to use [40].

#### Stress and anxiety among telehealth users

Attending antenatal appointments is an important part of the pregnancy journey for expectant parents [84]. A 2022 thematic analysis of 507 expectant parents conducted in the UK showed that parents experienced heightened anxiety due to reduced face-to-face contact during the pandemic, and that they felt telehealth was less personal and did not always adequately address their concerns [85].

Jongsma et al. [73] examined pregnant women’s experiences with remote monitoring for hypertensive disorders of pregnancy. Although their cohort was small (*N* = 52), some women reported stress arising from the results of home monitoring devices, while others found it reassuring [73]. Ghimire et al. also noted anxiety as one of the biggest barriers to participating in virtual antenatal care, and Konnyu et al. noted anxiety as a factor which favoured traditional care [48, 69].

Importantly, it seems increased anxiety can be overcome. Konnyu et al. reported that women who were initially sceptical of telehealth generally had their fears alleviated once engaging with this model of care [69]. Similarly, Nguyen et al. [86] identified through 25 semi-structured interviews that women had concerns regarding self-monitoring and accessibility of telehealth, but that these were alleviated by attending appointments with providers they already knew, highlighting the importance of continuity of care.

Furthermore, an increase in anxiety is not universal. In qualitative interviews with 18 pregnant women, Mehl et al. [74] found that participants who used telehealth could avoid stressors that were associated with in-person visits (e.g. travel, childcare, time off work) and thereby reduce anxiety. Similarly, in the 2017 BuMP feasibility trial, Hinton et al. [87] found that remote blood pressure monitoring reduced women’s health anxiety, particularly if they had previous experiences of hypertension or preeclampsia. In a cross-sectional study of 403 women, Mittone et al. [88] noted that education and income were positively associated with telehealth satisfaction, demonstrating the need to support women of disadvantaged backgrounds when accessing these services.

These studies highlight the importance of considering the potential for maternal anxiety when implementing telehealth services. This could be overcome by early education about its use, and reassurance about its safety.

#### Access to health services

Inadequate access to antenatal care is associated with adverse maternal and neonatal outcomes [10]. Inadequate access to care is significantly associated with social determinants of health, such as socioeconomic status, residential area, and education level [89]. There are concerns that telehealth may widen existing inequities, as these at-risk groups often also have limited technology literacy [90].

Generally, telehealth is shown to increase access to antenatal care. An Australian cross-sectional study found that pregnant women reported telehealth was more accessible than face-to-face, particularly for those in rural settings or with childcare responsibilities [91]. However, this study excluded women without access to a computer [91]. Studies from other settings have also reported improved access [48, 72, 92, 93]. Conversely, Osarhiemen et al. [94] identified that while telehealth did not mitigate inequities in access, it also did not widen existing disparities in the USA.

Improved access is not universal, however. Many reported barriers to telehealth implementation, such as Internet connectivity, lack of privacy, and low technology literacy, are more prevalent among already disadvantaged groups [75]. Morgan et al. [75] conducted a survey of pregnant women in the USA and found that 10% lacked the equipment necessary to complete their telehealth visits. Additionally, telehealth is not often covered by insurance to the same extent as face-to-face care, creating a significant financial barrier [95]. Hinton et al. [96] interviewed women and healthcare providers and further identified digital poverty; domestic violence; low literacy levels; sociocultural factors; and language background as barriers preventing equitable access to telehealth.

While telehealth has the capacity to improve access to antenatal care, it is important to consider inequities faced by disadvantaged groups during implementation so as not to widen existing disparities.

### Provider satisfaction

Telehealth is generally viewed favourably by providers. In their systematic review, Konnyu et al. included 674 healthcare providers and identified that they valued a more flexible, reduced-visit schedule; that they felt telehealth would be more convenient for pregnant women; and that it would allow more clinic time to be dedicated to high-risk pregnancies [69]. Hofman et al. [97] interviewed 56 maternity care providers, who reported telehealth to be feasible (94%), appropriate (80%), and acceptable (83%). Hargis-Villanueva et al. [98] saw that 89% of providers were highly satisfied with telehealth, and 72% would prefer to use it in the future.

Concerns about telehealth have also been raised. In their systematic review, Konnyu et al. identified the following concerns: inability to provide timely information that women may lack confidence in managing their pregnancies independently; and that reduced visits may compromise care and psychosocial need [69]. Hofmann et al. [97] identified further barriers: lack of equipment, inadequate clinic support, and poor image/sound quality. In semi-structured interviews with obstetric providers, Holman et al. [99] noted that some providers were also concerned that telehealth could widen existing health inequities.

Many of these themes mirror pregnant women’s concerns and highlight the need for clinician and patient education and support when implementing telehealth into models of care.

## Strengths and limitations

We did a narrative review instead of a meta-analysis so we could cover a broad range of topics. Whilst this is a narrative review, a rigorous and systematic search of the literature was completed. Over 7000 papers were screened, of which over 90 were deemed appropriate for inclusion in this review. We have provided a broad overview covering a wide range of topics, not just examining safety, but other important aspects of antenatal care — including consumer satisfaction, cost-effectiveness, and the various models of telehealth. We also focused on papers published between 2020 and 2023, making this review timely and contemporary.

This review does have some limitations. Most of the literature comes from high-income settings. Thus, the use of telehealth in low- and middle-income countries is not well-represented. Much of the included evidence was observational, due to a lack of recent randomised trials. Our conclusions may change in the future if this topic is revisited when more randomised trials are published. We conducted our systematic word search using the biomedical databases, MEDLINE and PubMed. It is therefore possible that papers listed in other databases may have been missed. However, given our review focuses on obstetrics, and that over 7000 records were identified from our search, it is likely that the vast majority of relevant evidence has been captured. Additionally, reference lists of included papers were searched to ensure that additional relevant papers were identified. Lastly, we note the number of participants was limited in many of the papers we reviewed, especially those examining adverse events. It is possible many studies so far have lacked the power to detect rare adverse outcomes caused by the use of telehealth technologies.

Furthermore, an important confounding factor is that many recent studies were done during the COVID-19 pandemic era where many countries had lockdown policies in place. In particular, many studies have compared the incidence of adverse pregnancy outcomes arising from telehealth done during the pandemic era to historic cohorts who birthed before the onset of COVID-19, thus results may have been affected by pandemic restrictions. Given these limitations, ongoing surveillance of health outcomes with the use of telehealth will be important to do.

## Conclusions

As telehealth becomes increasingly common in healthcare, it is important to understand how this can be translated into the obstetric context. Antenatal care has historically been delivered face-to-face, but the onset of the COVID-19 pandemic has facilitated a rapid and widespread shift towards telehealth.

Reassuringly, many studies examining the clinical safety of antenatal telehealth (including many done during the pandemic era) have not found an associated increase in adverse maternal or neonatal outcomes. However, larger cohort studies would be useful to exclude the possibility that telehealth increases the risk of rarer outcomes, such as stillbirth or maternal mortality. Telehealth also seems to be a cost-effective alternative to traditional care models, and possibly even cost-saving. Overall, surveys of women and antenatal care providers have shown high satisfaction with telehealth.

Although the evidence presented in this review has been largely positive, further research is required to elucidate the true impact of telehealth on antenatal care. It will be important for randomised trials to be conducted to strengthen the evidence base and provide certainty around this rapid change in practice. Further large studies are also required to assess rare outcomes, such as maternal and neonatal mortality, and to provide more clarity about trends that we are starting to see — including changing rates of caesarean section and preterm birth. Ongoing surveillance is also necessary to examine the effect of telehealth outside of the pandemic setting.

The landscape of antenatal care is changing. Potential advances in antenatal care should be embraced, for the benefit of pregnant women and their care providers. Telehealth may provide a valuable, cost-saving opportunity to expand the current antenatal care model, without sacrificing safety or consumer satisfaction.

### Supplementary Information

**Additional file 1: Table S1.** Search Strategy and Search Results – OVID Medline Database.

**Additional file 2: Table S2.** Search Strategy and Search Results – PubMed Database.

## Data Availability

Data sharing is not applicable to this article as no datasets were generated or analysed.

## Abbreviations

95% CI: 95% Confidence interval
ACOG: American College of Obstetricians and Gynecologists
COVID-19: Coronavirus disease 2019
HbA1c: Glycated haemoglobin
MD: Mean difference
NICU: Neonatal intensive care unit
OR/aOR: Odds ratio/adjusted odds ratio
RD: Risk difference
RR/aRR: Risk ratio/adjusted risk ratio
SD: Standard deviation
