# Supplementary material for: Telehealth in antenatal care: recent insights and advances
Source: BMC Med. 2023 Aug 30;21:332. doi: 10.1186/s12916-023-03042-y (PMC10470141; doi:10.1186/s12916-023-03042-y)
Supplement: Supplementary file 1 — Additional file 1: Table S1. Search Strategy and Search Results – OVID Medline Database. [file 12916_2023_3042_MOESM1_ESM.docx]

**Additional File 1: Table S1: Search Strategy and Search Results – OVID Medline Database**

| **Number** | **Search Term** | **Results** |
| --- | --- | --- |
| #1 | exp telemedicine/ OR exp telemetry/ OR exp monitoring, ambulatory OR (telemedicine OR telehealth OR teleultrasound OR teleobstetric* OR remote monitor* OR remote care).mp | 102,708 |
| #2 | exp obstetrics/ OR exp prenatal care/ OR exp maternal health services/ OR exp pregnancy/ OR (matern* OR prenatal* OR antenatal* OR antepartum* OR pregnan* OR obstetric* OR fetal).mp | 1,459,675 |
| #3 | #1 AND #2 | 2,944 |
