# Supplementary material for: Telehealth in antenatal care: recent insights and advances
Source: BMC Med. 2023 Aug 30;21:332. doi: 10.1186/s12916-023-03042-y (PMC10470141; doi:10.1186/s12916-023-03042-y)
Supplement: Supplementary file 2 — Additional file 2: Table S2. Search Strategy and Search Results – PubMed Database. [file 12916_2023_3042_MOESM2_ESM.docx]

**Additional File 2: Table S2: Search Strategy and Search Results – PubMed Database**

| **Number** | **Search Term** | **Results** |
| --- | --- | --- |
| #1 | telehealth OR telemedicine OR teleobstetrics OR teleultrasound OR tele-health OR tele-medicine OR tele-obstetrics OR tele-ultrasound OR remote monitoring OR remote care | 91,719 |
| #2 | obstetrics OR prenatal OR antenatal OR maternal health OR pregnancy OR antepartum OR fetal | 1,618,729 |
| #3 | #1 AND #2 | 4,104 |
